# Alterations of Intrinsic Brain Connectivity Patterns in Depression and Bipolar Disorders: A Critical Assessment of Magnetoencephalography-Based Evidence

**DOI:** 10.3389/fpsyt.2017.00041

**Published:** 2017-03-17

**Authors:** Golnoush Alamian, Ana-Sofía Hincapié, Etienne Combrisson, Thomas Thiery, Véronique Martel, Dmitrii Althukov, Karim Jerbi

**Affiliations:** ^1^Department of Psychology, University of Montreal, Montreal, QC, Canada; ^2^Department of Computer Science, Pontificia Universidad Católica de Chile, Santiago de Chile, Chile; ^3^Interdisciplinary Center for Neurosciences, School of Psychology, Pontificia Universidad Católica de Chile, Santiago de Chile, Chile; ^4^Center of Research and Innovation in Sport, Mental Processes and Motor Performance, University Claude Bernard Lyon I, University of Lyon, Villeurbanne, France; ^5^Brain Dynamics and Cognition, Lyon Neuroscience Research Center, INSERM U1028, UMR 5292, University of Lyon, Villeurbanne, France; ^6^Department of Computer Sciences, National Research Institution Higher School of Economics, Moscow, Russia; ^7^MEG Center, Moscow State University of Pedagogics and Education, Moscow, Russia; ^8^Centre de recherche de l’Institut universitaire en santé mentale de Montréal, Montreal, QC, Canada

**Keywords:** magnetoencephalography, connectivity, resting-state, psychiatry, depression, bipolar disorder, mental illness, oscillations

## Abstract

Despite being the object of a thriving field of clinical research, the investigation of intrinsic brain network alterations in psychiatric illnesses is still in its early days. Because the pathological alterations are predominantly probed using functional magnetic resonance imaging (fMRI), many questions about the electrophysiological bases of resting-state alterations in psychiatric disorders, particularly among mood disorder patients, remain unanswered. Alongside important research using electroencephalography (EEG), the specific recent contributions and future promise of magnetoencephalography (MEG) in this field are not fully recognized and valued. Here, we provide a critical review of recent findings from MEG resting-state connectivity within major depressive disorder (MDD) and bipolar disorder (BD). The clinical MEG resting-state results are compared with those previously reported with fMRI and EEG. Taken together, MEG appears to be a promising but still critically underexploited technique to unravel the neurophysiological mechanisms that mediate abnormal (both hyper- and hypo-) connectivity patterns involved in MDD and BD. In particular, a major strength of MEG is its ability to provide source-space estimations of neuromagnetic long-range rhythmic synchronization at various frequencies (i.e., oscillatory coupling). The reviewed literature highlights the relevance of probing local and interregional rhythmic synchronization to explore the pathophysiological underpinnings of each disorder. However, before we can fully take advantage of MEG connectivity analyses in psychiatry, several limitations inherent to MEG connectivity analyses need to be understood and taken into account. Thus, we also discuss current methodological challenges and outline paths for future research. MEG resting-state studies provide an important window onto perturbed spontaneous oscillatory brain networks and hence supply an important complement to fMRI-based resting-state measurements in psychiatric populations.

## Introduction

### Background

Over the last decade, research on the human brain has experienced an important shift in paradigm; the functional investigation of neuronal activity has moved from studying local mechanisms toward large-scale network organization. Unavoidably, this change in the examination of neural connectivity has reached the field of psychiatry. Until recently, most connectivity studies in psychiatric patients were predominantly carried out using functional magnetic resonance imaging (fMRI). The findings from these studies generally indicate the presence of structural and/or functional abnormalities linked to the diseases [e.g., Ref. (1–3)]. Moreover, irregularities are not only observed during cognitive tasks, when subjects are engaged in a sensory, cognitive, or emotional task, but also during rest, when subjects are asked to lay still in the scanner and let their mind wander. The trend in the field of neuroimaging, toward the study of this so-called resting state, has strongly contributed to unveiling intrinsic properties of brain disorders (4–7). Yet many questions about the neurophysiological bases of resting-state alterations remain unanswered.

A parallel stream of research explores the physical connections between brain regions by assessing structural connectivity with magnetic resonance imaging (MRI) using diffusion tensor imaging (DTI) and fractional anisotropy. These techniques allow the examination of white matter integrity and fiber tract organization and are thereby able to reveal anatomical disruptions of long-range structural connections (8). However, DTI is principally useful for pathologies for which we know of preexisting structural anomalies and less so for illnesses without obvious disruptions in connectivity (4, 9). Furthermore, although fMRI is promising for the investigation of the spatial organization of the cortex, it is limited by its temporal resolution and by the fact that it is an indirect measure of neural activity. Moreover, because it measures the brain’s hemodynamic responses, fMRI is useful to study slow activity fluctuations (i.e., <0.1 Hz), but is unable to capture brain activity patterns at higher frequencies. Consequently, neuroimaging methodological developments and studies of the past 10 years have been reflective of the scientific community’s appreciation of the importance of electrophysiology for our understanding of network connectivity (10, 11). This change is portrayed by the flux in research employing electroencephalography (EEG), intracranial electroencephalography (iEEG), and magnetoencephalography (MEG), three tools with excellent temporal resolution. Specifically, a spotlight has been shined on the behavior of local and long-range synchronized brain oscillations in healthy cognition and, also, as potential markers for altered neural connectivity in (psychiatric) diseases (12–15).

When small neighboring neuronal populations synchronize their oscillations, local assemblies are forged, and coupling among these small assemblies can bridge distant areas (creating long-range connections) (10). Disruptions in this mechanism could unravel a number of neuropathologies and psychopathologies. Neuronal synchronizations are thought to operate on short-time scales, and changes in spectral power are optimally detectable by electrophysiological recordings. Thus, we can examine neural network connectivity patterns by measuring the electrophysiological activity of two or more brain regions of interest (ROIs) using EEG, iEEG, or MEG (16). Of increasing interest, MEG (17) has emerged as a valuable, non-invasive tool to assess local and long-range modulations of synchronized neural activity in humans [e.g., Ref. (14, 18–24)].

Although EEG permits the probing of large-scale networks, with high temporal resolution, MEG has a number of advantages. For instance, the magnetic signal that is captured by MEG is less distorted by brain tissue and skull than the electrical field detected by EEG. In addition, MEG source reconstruction methods can provide valuable spatial information to better characterize neural network modulations. Finally, in the context of clinical research, and more specifically in psychiatry, the fast and easy setup of the MEG system is likely to be less unnerving for patients than the lengthy procedure of EEG. Taken together, exploration of the potential of MEG in psychiatry is an important endeavor that could lead to better understanding of psychopathology. Further details about the technical aspects of MEG have been overviewed elsewhere [e.g., Ref. (18, 20, 23, 25)].

### Purpose of This Review

Despite being the object of a thriving field of clinical research, the investigation of intrinsic neural network alterations in psychiatric illnesses is in its early days and is predominantly conducted using fMRI or EEG. The recent contributions and future promise of MEG in this field are not fully recognized and valued. In this article, we review recent findings in MEG resting-state connectivity within two mood disorders: major depressive disorder (MDD) and bipolar disorder (BD). Most importantly, this review provides a critical assessment of currently employed methods and outlines important limitations that need to be considered in future resting-state MEG studies of mood disorders.

### Important Concepts and Terminology

#### Resting-State Networks

When subjects are asked to lay or sit still in an MRI, PET, EEG, or MEG setup and to let their minds wander, the activity that arises is one that speaks of the fundamental organization—or disorganization—of the brain [e.g., Ref. (26–29)]. Resting states can be categorized into several networks (on the order of 7 ± 1): the sensorimotor network, the primary and extrastriate visual network, the auditory network, lateralized frontoparietal networks, the temporoparietal network, the central executive network (CEN), and the, most extensively studied, default mode network (DMN) (27, 30–35). The DMN englobes primarily the medial prefrontal cortex (mPFC), the posterior cingulate cortex, and the precuneus cortex, as well as the inferior parietal cortex, the lateral temporal cortex, and the subgenual anterior cingulate cortex (ACC) (27, 34). A large amount of evidence shows the DMN to be deactivated when one is engaged in a cognitive or sensorimotor task and active during rest or meditative tasks (7, 36–39). Of particular interest, disruptions in this network have been linked to the occurrence of psychopathological symptoms (e.g., depressive, manic, or psychotic episodes) (27, 34, 40–42).

#### Anatomical, Functional, and Effective Connectivity

The literature on neural network connectivity, particularly graph theory, suggests that the purpose of a node is guided by how it is connected to other nodes in a given network and that its function is a consequence of the action of its integral network (43). Hence, when resting-state activity is observed for a few minutes, the spontaneous oscillatory behaviors form consistent and reliable functional networks [e.g., Ref. (44)]. The efficiency of the connections within and between these networks appears to rely on at least two main factors: epigenetics and experience (45). The first factor pertains to the interaction between genes and the growth of brain structures, whereas the second pertains to intrinsic neural activity and activity-dependent changes in synaptic strength (e.g., learning) elicited by a person’s interaction with their environment.

Three types of connectivity are generally examined: anatomical, functional, and effective. First, anatomical connectivity pertains to the physical connection between brain regions. It is typically examined using MRI-based DTI analysis of white matter, axonal, and tracts (46). Second, functional connectivity is used to describe the statistical dependency of time-series activity arising from two brain areas (9, 47, 48). It can be measured using linear and non-linear tools such as correlations, coherence, phase-lag index, and mutual information (49–52). It is important to note that although evidence from both human and animal studies shows a close relationship between structural and functional connectivity (5, 53–55), direct anatomical linkage is not necessary for functional connections to take place (54, 56). Finally, effective connectivity speaks of the direct or indirect influence of one brain system on another based on neuronal coupling (9, 44) and can be measured using metrics such as Granger causality and direct transfer functions (57).

In this review, we focus on functional connectivity abnormalities across MDD and BD.

#### Local Power Modulations vs Long-Range Interareal Connectivity in MEG and EEG

It has been proposed that local and long-range neural synchrony patterns speak of the inherent organization of the brain (43, 58), and thus, an exploration of oscillatory rhythms could help us understand the fundamental neural functioning of different populations. However, confusion can emerge for investigators who are new to the discussion on neural network connectivity. This confusion is entangled in the lack of consistency in the vocabulary employed to describe the two different processes of local and long-range synchrony. It has been argued that when neural populations synchronize, it is a phenomenon that expands across multiple temporal and spatial scales, from local integration of information within the areas that specialize in the same functions to long-range connections that connect different modalities of an object (10). Generally speaking, *local synchrony* is what is captured by power estimation from a single brain signal (e.g., data from one channel or cortical source). By contrast, *long-distance synchrony* is captured by estimating the coupling between data from two brain signals.

Specifically, *local power modulations* of a neural population reflect the activity of a small spatial area of neurons on the order of 1 cm [based on experiments in visual networks, e.g., Ref. (59)]. The measure of spectral power is taken as a reflection of the amplitude of oscillations at different frequencies (60). Neurophysiological studies have underlined the importance of examining local synchronization to observe the different types of information that are carried by different frequency bands (10, 61). As for *long-range connectivity*, it reflects the functional coordination and synchronization of time series from two brain regions that may or may not have direct structural linkage (e.g., through myelinated white matter tracts). This type of connectivity bridges brain areas between and within different neural networks (43, 58, 60, 62).

Although both local and long-range synchrony can be measured at sensor and source levels during EEG and MEG studies, caution should be taken when measuring the statistical or coherence differences between two recording (sensor) sites. Indeed, what may first be thought to be coordinated time series reflecting connectivity between two brain areas (63) may in fact be spurious coupling arising from volume conduction or field spread [e.g., Ref. (48, 64)]. Different methods have been proposed to overcome this challenge. A solution that can be applied to reduce the impact of this linear mixing limitation is to use coupling measures that are not overly affected by field spread and perform the connectivity estimations in MEG source space [cf Ref. (48, 52, 65, 66)] [see Contrasting Controls and Patients: Differences in Artifacts and Signal-to-Noise Ratio (SNR)].

## Abnormal Connectivity in Psychiatric Disorders: Where Do We Stand?

The following section provides a brief and non-exhaustive multimodal overview of the rapidly increasing body of neuroimaging research that links psychiatric disorders with pathological alterations in neuronal connectivity, in line with previous work that overviewed network connectivity in SZ (60, 67) and depressed patients (34) across different neuroimaging modalities. Because of the current effervescent nature of this field, an in-depth account of the neuronal network dysfunction in mental illness is beyond the scope of this review. Instead, we will focus on findings that are particularly relevant to past, current, and potentially future resting-state MEG investigations. With this in mind, we first describe recent resting-state EEG and fMRI evidence that suggests dysfunctional intrinsic neural communication in MDD and BD.

### Major Depressive Disorder

With over 100,000 scientific papers on PubMed, depression is the most common and the most studied psychiatric illness in humans. MDD is characterized by features such as low mood and/or a loss of interest in daily activities for an extended amount of time and, typically, involves ruminative, self-referential thoughts (68). A lifetime prevalence of 11.3% has been reported in Canada (69) and 16.2% in the United States (70), whereas across the world, it is estimated that 350 million individuals suffer from depression (71).

Although a number of impactful task-based studies have explored alterations in oscillatory synchronizations [e.g., Ref. (72–74)], the following subsections will focus on resting-state fMRI and EEG studies in MDD population.

#### fMRI Resting-State Connectivity Findings in MDD

Given the DMN’s role in self-referential behaviors (7, 27), this network, particularly the mPFC, is recurrently noted as a region important for the discrimination of depression from normal population (34, 75–77). Specifically, fMRI studies show depressed patients to display increased connectivity between certain nodes of the DMN [for instance, between the subgenual ACC and the posterior cingulate cortex (78)], which could be detrimental to cognitive processes (4, 79). These findings are supported by reviews that also highlight enhanced patterns of connectivity within these areas of the DMN (3, 80). In addition, studies have observed altered connectivity between nodes of the DMN and the nodes of the CEN. For instance, one study found enhanced connectivity between the dorsolateral prefrontal cortex (PFC) and the subgenual ACC (81), whereas a review found decreased connectivity between the inferior/superior parietal DMN and dorsal CEN (3).

Moreover, a number of original studies and reviews have reported atypical functional connectivity within areas key to emotional processing. Indeed, limbic regions [amygdala, insula (82, 83)], parts of the DMN [mPFC (77, 83)], and long-range connections between the DMN and the limbic system [e.g., thalamus and posterior cingulate cortex (79)], as well the CEN and the limbic system [e.g., dorsolateral PFC and amygdala (84–86)], appear reduced in patients compared to controls. However, connectivity between the saliency network (i.e., anterior insula, dorsal ACC) and the anterior DMN (34), as well as between the insula and the amygdala (87) seems enhanced in depressed individuals when compared with controls.

All in all, within and between network connectivity of the DMN and limbic system appear to be altered in MDD patients, particularly with respect to projections involving the subgenual ACC. The atypical resting-state organization of their brain appears to correlate with their cognitive and emotional symptoms (88).

#### EEG Resting-State Connectivity Findings in MDD

##### EEG Power Modulations (Local Synchronization)

Examinations of local synchronizations in depressed populations have consistently found low-frequency bands (<20 Hz) to display enhanced power and coherence across most brain regions (3, 89–93). However, power modulations in higher frequency band (>30 Hz) do not seem to be a discriminative factor to differentiate the resting-state of depressed and control subjects [e.g., Ref. (3)].

##### EEG Connectivity (Long-Range Synchronization)

Similar to the findings from fMRI, EEG studies have found disruptions and asymmetrical connectivity patterns within the frontal lobe of MDD patients in theta (5–7 Hz) and alpha (8–13 Hz) frequency bands compared with healthy control subjects (94, 95). Treatment-based EEG studies established once more the importance of the subgenual ACC. Indeed, depressed patients appear to show enhanced connectivity within nodes of the DMN and between nodes of the DMN and the CEN (3, 92). Specifically, enhanced connectivity in alpha frequency band (8–12 Hz) between the subgenual ACC and left mPFC is observed before antidepressant treatment, which switched into enhanced connectivity in beta frequency band (12.5–20 Hz) between the subgenual ACC and right mPFC after antidepressant treatment, thus underlying the recurrent asymmetrical connectivity patterns observed in MDD patients’ frontal lobe (92, 95). Moreover, it has been suggested that alterations in frontotemporal connectivity in delta/theta (1–8 Hz) frequency range could be used a marker to predict responders and non-responders to antidepressant medication (i.e., selective serotonin reuptake inhibitor), with hyperconnectivity between these areas being associated to poorer response (96).

The observations discussed above are consistent with insights achieved using deep brain stimulation (DBS) in MDD patients. Clinical trials with DBS have targeted the overactive subgenual ACC and the thalamocortical pathway, for the treatment of severe depression (97, 98). Although the success of such surgical procedure is still debated [(99–103)], DBS involves the implantation of intracranial electrodes that allow a rare window into the circuitry of MDD.

### Bipolar Disorder

Bipolar disorder is a functionally debilitating disorder. The main categories of BD are BD-I and BD-II, which are characterized by a combination of manic episodes and depression and by hypomania episode(s) and depressive symptoms, respectively (68). A lifetime prevalence of 2.6% has been reported in Canada (69) and around 4% (across all types of BD) in the United States (104), while across the world, it is estimated that 60 million individuals suffer from BD (71).

Task-based fMRI/EEG studies that have explored connectivity patterns in BD population have found synchronization alterations that differentiate them from healthy individuals (105–107). However, in this section, we will explore the research that has studied BD patients during a resting-state condition.

#### fMRI Resting-State Connectivity Findings in BD

Multiple fMRI studies have attempted to untangle the connectivity anomalies observed in these patients, with somewhat contradicting results. Indeed, depending on the analytical method used to extract connectivity, whether it be independent component analysis (ICA) or seed-based/ROIs, wavering conclusions have been made on BD patients’ corticolimbic connectivity patterns [e.g., Ref. (108)].

Although DMN activity has been closely linked to mind wandering and interoceptive thoughts (7, 40), it has also been shown to be germane to social cognition, which is known to be impaired in psychiatric disorders (109–111). A number of fMRI studies have summarized that most bipolar patients and their unaffected relatives have decreased connectivity within the nodes of the DMN (36, 37, 112), between the mPFC and the insula compared with controls (113). Hyperconnectivity has been noted between the DMN and the CEN (114), the DMN and the temproparietal network (36, 37), and the DMN and the visual and the auditory networks (36, 37, 115) compared to controls. Finally, the predominant result of this research in BD, based on reviews of fMRI and DTI studies, shows altered connectivity between parts of the DMN (e.g., mPFC) and the limbic system [e.g., amygdala (42, 85, 108, 114, 116–118)].

With respect to the ventrolateral PFC, contradicting findings have been reported, with some estimating enhanced connectivity with the amygdala (108, 118), while others diminished (119) compared to controls. In addition, a number of studies have investigated the influence of psychotic symptoms on network connectivity patterns during resting state. For instance, one paper observed hypoconnectivity within the PFC and between the dorsolateral PFC (CEN) and amygdala (120), which was predominantly present in BD patients who presented with a history of psychosis, and not in non-psychotic BD or controls.

All in all, models proposed by many researchers suggest that disruption between these keys areas, particularly connectivity involving the amygdala, underlie the occurrence of manic symptoms and inefficient emotional management (108, 118).

#### EEG Resting-State Findings in BD

##### EEG Power Modulations (Local Synchronization)

Electroencephalography analyses of local synchronization have predominately reported group differences in the frontal region and the cingulate cortex. Examinations in modulations of local oscillatory behavior in the frontal cortex show enhanced power in alpha (8–13 Hz) (121), beta, and gamma (122, 123) frequency bands compared to controls. In the cingulate cortex, low-frequency bands, theta and alpha, had decreased power (122, 124, 125), while higher frequency bands, beta (15–30 Hz) and gamma (30–50 Hz), displayed increased power compared with controls (122, 123).

##### EEG Connectivity (Long-Range Synchronization)

With respect to long-range connectivity, an EEG investigation noted decreased connectivity in the alpha (8–12 Hz) frequency band within frontocentral and centroparietal neural network connections of patients compared with healthy controls (124). However, more studies are needed to confirm this finding and increase the specificity of the affected regions. The accumulated evidence from multiscale modalities (e.g., fMRI and EEG) indicates that individuals affected by BD display atypical local and long-range connectivity patterns within the nodes of the DMN and between the PFC and the amygdala.

Taken together, the fMRI and EEG findings in MDD and BD patients indicate that these populations may have difficulties processing and transferring information economically that can be detectable as connectivity anomalies between and within resting-state networks. Research into pathological alterations of resting-state activity provides critical insights into large-scale network dynamics, which complement key findings that continue to emerge from task-based studies (126). In the next section, we will overview the results of MEG studies that examined resting-state connectivity and alterations in frequency band modulations within the MDD and BD. We will also examine the overlap in connectivity findings that has been reported across different resting-state neuroimaging modalities.

## Resting-State MEG Connectivity and Mood Disorders: What Do We Know?

Although still in its early days, MEG has led to important clinical insights in numerous brain disorders and has become a promising tool for clinical and translational research in psychiatry (14, 127). In the following, we will focus specifically on MEG contributions to elucidate resting-state alterations in mood disorders.

### Major Depressive Disorder

A hierarchical approach was taken to isolate the keywords that were most appropriate for this review. First, we began with “MEG + condition,” where condition was the general term for either MDD (i.e., depression) or BD (i.e., bipolar). We then refined this search by adding the term “resting” to capture studies that included a resting-state paradigm. Finally, we used the term “connectivity” to capture publications that also evaluated long-range synchronization, as resting state alone, could potentially refer to power analyses. Various combinations of these terms, as well as cross-referencing, were employed to ensure that all studies investigating MEG resting-state local power and long-range synchrony in MDD and BD patients were considered.

Here, a PubMed search using the key words “MEG + connectivity + depression” resulted in 16 hits, 12 of which were, however, unrelated to our topic of interest. Another search of the keywords “MEG + depression + resting” yielded 12 studies, with only 2 studies being relevant additions to this review. Among the articles that were found, the final count of scientific articles included in this article is 5.

#### Altered Resting-State MEG Power Patterns in MDD

The recent article by Jiang et al. (128) compared the oscillatory activity of MDD patients with those of age- and education-matched control subjects. Depression correlated with power decrease in theta (4–8 Hz) and alpha (8–14 Hz) frequency bands in the frontal and parietal areas, respectively, as well as with enhanced power in beta frequency band (14–30 Hz) oscillations in the DMN. Similar to EEG findings [see EEG Power Modulations (Local Synchronization)], no significant difference was found between the two populations across higher frequency bands [>30 Hz (128)].

Moreover, Li et al. (129) examined MEG signals in treatment-resistant MDD individuals who received 10 daily repetitive transcranial magnetic stimulation (rTMS) in the region of the dorsolateral PFC for two consecutive weeks. The authors normalized the spectral amplitude of five frequency bands (delta, 2–4 Hz; theta, 4–8 Hz; alpha, 8–13 Hz; beta, 13–30 Hz; and gamma, 30–50 Hz) by the mean power across all bands to obtain a relative amplitude index for each oscillatory band. Moreover, they measured frontal alpha asymmetry (FAA) in all their subjects, as FAA had been previously associated with symptom severity in depression (130). This article, however, found no significant difference between patient and control subjects in terms of FAA, similar to the inconclusive results of previous electrophysiological studies on FAA, e.g., Ref. (15, 131).

#### Altered Resting-State MEG Connectivity Patterns in MDD

Table 1 summarizes the details of the four studies on MEG resting-state connectivity in MDD patients. The article by Nugent et al. (132) demonstrated that resting-state networks are altered in MDD patients compared with controls in beta frequency band (14–30 Hz). Specifically, based on temporal ICAs and correlations in source space, they found patients to have altered connectivity between nodes of the DMN and the limbic system. Of note, long-range connectivity between the subgenual ACC and the hippocampus was diminished in patients (132). Moreover, the authors observed MDD patients to show enhanced connectivity between the right insular-temporal region and parts of the limbic system (i.e., amygdala, thalamus), and the left insular-temporal region and the angular gyrus in the parietal lobe and the precentral gyrus, which is part of the posterior region of the frontal lobe (132).

**Table 1:** **MEG resting-state connectivity studies in major depressive disorder**.

Reference	Frequency range	Methods	Patients	Controls	Main findings
(129)	Delta: 2–4 Hz Theta: 4–8 Hz Alpha 8–13 Hz Beta: 13–30 Hz Gamma: 30–50 Hz	Frontal alpha asymmetry and voxel-based partial correlation to examine connectivity in prefrontal-thalamic circuit (based on PET) Sensor-space analysis 3 min eyes open	30 MDD received rTMS, 6 males 17 responders: mean age: 51.9 ± 10.5 13 non-responders: mean age: 50.1 ± 6.2	50 controls 14 males mean age: 49.1 ± 7.0	MDD appeared to have impaired prefronto-thalamic functional connections compared to controls. rTMS resolved this pattern in those who responded to treatment after 2 weeks of treatment at 10 Hz in their dorsolateral PFC
(132)	14–30 Hz	Correlation Source-space analysis 4.17 min eyes closed	33 MDD 22 males Mean age: 42.8 ± 9.9	19 controls 11 males Mean age: 39.3 ± 6.5	Patients had reduced correlations between the subgenual ACC and hippocampus in a network with primary nodes in the precentral and middle frontal gyri. Patients showed increased correlations between insulotemporal nodes and amygdala compared to controls
(133)	14–30 Hz	Correlation Source space 4.17 min eyes closed	13 MDD 11 males Mean age: 45.0 ± 13.2	18 controls 12 males Mean age: 39.0 ± 7.3	Patients displayed enhanced connectivity between insulotemporal areas and amygdala that were reduced to normal levels after ketamine treatment
(134)	Delta: 2–4 Hz Theta: 5–7 Hz Alpha: 8–12 Hz Beta: 15–29 Hz Gamma: 30–59 Hz	Magnitude-squared coherence. Seed: dorsolateral PFC Source space 6 min eyes closed	5 MDD received TMS, 1 non-responder	n/a	Symptom improvement by 10 Hz rTMS increased connectivity between dorsolateral PFC and amygdala, and dorsolateral PFC and pregenual ACC in delta band. rTMS decreased connectivity between dorsolateral PFC and subgenual ACC

*Overview of MEG resting-state studies examining changes in long-range connectivity patterns in subjects with MDD*.

*MDD, major depressive disorder; rTMS, repetitive transcranial magnetic stimulation; PFC, prefrontal cortex; ACC, anterior cingulate cortex; MEG, magnetoencephalography*.

In a follow-up MEG study (133), the same authors sought out to examine the effect of ketamine on long-range synchronizations in MDD patients. The source-space connectivity patterns that were uncovered were similar to the disrupted areas found in their earlier article (132). In the beta frequency band (14–30 Hz), 0.5 mg/kg ketamine restored the abnormal hyperconnection between amygdala and insula-temporal regions to normal levels. Interestingly, the authors noted that ketamine appeared to decrease all connectivity patterns across all the regions of the brain, regardless of the subjects’ baseline activity.

Pathak et al. (134) recently used the magnitude-squared coherence to estimate long-range connectivity in depressed individuals before and after rTMS in the dorsolateral PFC at 10 Hz. Their source-space findings (*via* minimum-norm estimate) reveal that symptom improvement after 4 weeks of treatment correlated with changes in the connectivity within the DMN. Post-TMS, MDD patients found increased coherence between the dorsolateral PFC and the amygdala and the pregenual cingulate cortex in the delta frequency band, as well as decreased coherence in the gamma band between the dorsolateral PFC and the subgenual ACC before treatment (134). The findings of this article could imply that baseline connectivity patterns in MDD involve diminished coherence between dorsolateral PFC-amygdala and dorsolateral PFC-pregenual cingulate cortex, along with enhanced coherence between dorsolateral PFC-subgenual ACC. Moreover, the outcome of this study underlines the importance of analyzing and reporting the type of treatment received by patients as it directly affects the neural network organization.

Finally, Li and colleagues’ (129) longitudinal study explored connectivity in alpha frequency band oscillations in the PFC (*via* MEG) and glucose metabolism in the thalamus (*via* PET), which is typically underactive in MDD patients. For the analysis of PFC and thalamus connectivity, patients were divided into binary categories of responders and non-responders to 2-week treatment of rTMS at 10 Hz. Patients were categorized based on their symptom ratings on the Hamilton Depression Rating Scale in the eighth week of the study. This type of antidepressant treatment was able to rescue the disrupted functional connection in responders 14 weeks after the start of the study, while this did not succeed in non-responders. Thus, according to the authors, their sensor-space finding could be seen as additional evidence that the strength of prefrontothalamic connectivity could be an index of depressive symptoms, as previously observed in fMRI studies (79).

The main finding of these MEG papers speak of altered long-range connectivity between the DMN and the CEN. In particular, the resting-state MEG literature supports previous fMRI studies that have demonstrated the implication of the subgenual cingulate cortex, the dorsolateral PFC, and the thalamus in illness severity and symptomatology in MDD [e.g., Ref. (79)]. Importantly, the subgenual cingulate cortex is typically targeted for DBS and rTMS in treatment-resistant depressed individuals and is thus critical to the understanding of their resting-state neural networks (97, 98, 135, 136).

#### Relationship to Task-Based MEG Findings

This section explores task-based MEG studies that corroborate connectivity results from resting-state MEG studies in MDD.

A number of studies have investigated the long-range connectivity patterns that emerge during affective and cognitive tasks in psychiatric patients. Among their findings, the diminished long-range synchronization between the dorsolateral PFC and the amygdala, observed recently in resting-state MEG by Pathak et al. (134), was also observed by Lu et al. (137). Indeed, the authors explored effective connectivity within the prefrontal-limbic system circuit using dynamic causal modeling analysis (137). During the affective task, subjects viewed 3-s clip of faces who were eating, neutral, happy, or sad and then indicated by button-press if the expression was sad or not. Under the most optimal model, patients had decreased connectivity from the dorsolateral PFC to the amygdala compared with controls. The authors hypothesized that this could explain part of the dysfunction observed in MDD patients with respect to the integration of both affective and cognitive information for overt behavior.

Other connectivity alteration in MDD patients have also been noted in task-based MEG studies. Specifically, a measure of wavelet coherence has shown enhanced connectivity between the ACC and the amygdala in the gamma (30–48 Hz) and in the delta (below 4 Hz) frequency bands (138). Moreover, enhanced connectivity between the amygdala and the inferior frontal gyrus, as well as between the amygdala and the ACC, in patients, was found to be highly discriminative features during the aforementioned affective task to differentiate MDD and control subjects (139). These connectivity alterations have yet to be observed in resting-state MEG findings. It may be the case that these differences in long-range synchronizations are due to the nature of the task or, rather, that they are best detected by these emotion-based paradigms.

Finally, Salvadore et al. (140) used the widespread working-memory task, N-back, to investigate how the connectivity patterns of MDD patients change before and after a single ketamine infusion. During this task, subjects were asked to keep in mind a stimulus (number between 1 and 4) and to respond when it was matched to the observed stimulus either right away or one or two trials previously. By using source-coherence analysis, the authors observed that the connectivity strength between the pregenual ACC and the left amygdala correlated negatively with the effect of treatment. Indeed, stronger coherence between these two regions prior to ketamine infusion correlated with improvement in symptoms. Similar findings about altered long-range synchronization between the subgenual ACC and the amygdala have been observed in intracranial EEG/DBS studies (97, 98). Given the reoccurring report of involvement of these brain regions, future resting-state MEG studies could clarify whether this pattern of connectivity alteration pertains to the nature of the task or to the intrinsic neural organization in MDD.

#### Strengths and Limitations of Resting-State MEG Studies in MDD Population

An important limitation that connectivity studies might display is that of being conducted at sensor level rather than source level. Although most of the reported MDD studies were conducted in source space, the article by Li et al. (129) was in sensor space, where only 26 gradiometers (out of a possible 306 channels) from the frontal region were used. However, the article had a major strength of employing a multimodal approach to studying connectivity (MEG, PET, and TMS), which included an anatomical T1 from MRI to obtain anatomical precisions. This allowed access to a richer set of information than what is provided using a single neuroimaging tool.

Next, exploring specific frequency bands can also be a limitation. Indeed, in the article by Nugent et al. (132), only the beta band frequency range was explored, while Li et al. (129) reported only on alpha frequency band oscillatory behavior. A strength of Nugent et al. (132) is that the authors took additional quality control steps (to verify the reliability of their ICA estimates), they tested the reliability of their results by comparing it with a second data set (unmatched groups) and, importantly, they included unmedicated MDD subjects. By doing so, the authors allowed to examine intrinsic connectivity prior to pharmaceutical effects.

Furthermore, the metric of coherence and correlations can raise questions about the spatial accuracy of the long-range synchronizations observed due to potential field spread effect (see Challenges, Pitfalls, and Methodological Recommendations for Future Studies for further details).

Finally, small sample size can be problematic in the interpretation of findings. In their recent study, Nugent et al. (133) explored the effect of ketamine on long-range synchronization and on symptoms scores as assessed by the Mania and Depression Rating Scale in a subset of patients from their previous study (132). However, this was performed in a small number of subjects. Non-parametric statistical tests were applied to compensate for their cohort of patients. Similarly, while Pathak et al.’s (134) study was important to evaluate the longitudinal effect of repetitive TMS on neural network connectivity, the small sample of patients (*n* = 5) and lack of multiple comparisons put the findings of this paper at risks of type I error, a fact acknowledged by the authors. While it is important to evaluate promising treatments, it would be interesting to evaluate its effect on connectivity patterns in a larger cohort of patients to increase reliability.

### Bipolar Disorder

A PubMed search of the key words “MEG + connectivity + bipolar” resulted in no findings. However, a search of the key words “MEG + bipolar + resting” yielded three studies, one of which was an EEG study (already discussed in fMRI Resting-State Connectivity Findings in MDD).

#### Altered Resting-State MEG Power Patterns in BD

Al-Timemy et al. (141) were able to successfully classify BD and control populations using MEG resting-state spectral features within the delta (1.5–4 Hz), theta (4–8 Hz), alpha (8–13 Hz), beta (13–30 Hz), and gamma (30–40 Hz) frequency bands. Relative power modulations in delta and theta frequency bands in the posterior region of the brain were observed to be significantly different between patients and healthy controls (141). However, the direction in these differences was not specified. The authors of this study also explored median frequency (MF), described as the frequency that divides the area under the curve of the power map (1.4–40 Hz) into two. Their analysis found MF of BD patients to range between 9.92 and 12.54 Hz depending on the examined brain region. Furthermore, unlike healthy control subjects who demonstrated a positive correlation between their MF and age, BD patients had a negative correlation between MF and age (141).

#### Altered Resting-State MEG Connectivity Patterns in BD

Table 2 summarizes the details of the relevant study that have been published on BD. Chen et al. (142) had an interesting, although small, pool of euthymic (no overt depressive or manic symptoms) BD-I patients that was compared with matched healthy controls. The authors focused on the frontal cortex activity, and thus, oscillatory modulations in only 11 of their 306 MEG channels were reported. Their spectral analysis across pairs of channels was performed using a derivative of the similarity index (SI) framework used by Arnhold and colleagues (143). Differences between patients and controls were noted based on global SI of channel pairs: patients displayed an increase in the synchronization of delta band (2–4 Hz) frequencies and a decrease in beta band (12–24 Hz) frequencies within nodes of the frontal cortex (142). While there is a number of MEG studies that have examined alterations of spectral power in BD during tasks [e.g., Ref. (144, 145)], as it stands, and to our knowledge, the article by Chen et al. (142) is so far the only resting-state MEG study that has evaluated the functional connectivity in BD population.

**Table 2:** **MEG resting-state connectivity studies in BD**.

Reference	Frequency range	Methods	Patients	Controls	Main findings
(142)	Delta: 2–4 Hz Theta: 4–8 Hz Alpha:8–12 Hz Beta: 12–24 Hz	Similarity index; using 11 sensors from the frontal lobe Sensor space 2 min eyes closed	10 euthymic BD-I 5 males Mean age: 32.5 ± 10.3	10 controls: 5 males Mean age: 32.2 ± 11.6	Increased synchronization of δ frequency oscillations and decreased synchronization of β frequency oscillations in the frontal lobe in BD compared to controls

*Overview of MEG resting-state studies examining changes in long-range connectivity patterns in subjects with BD*.

*BD, bipolar disorder; BD-I, bipolar disorder type I; MEG, magnetoencephalography*.

#### Relationship to MEG Task-Based Studies

To the best of our knowledge, no MEG task-based study has explored long-range synchronizations in BD. However, EEG resting-state studies, such as one by Kim et al. (124), have observed disrupted connections within the PFC of BD patients compared with controls. Future MEG studies, with and without tasks, could help elucidate more specific neural network patterns that are either specific to the neural organization of BD patients, or, alternatively, to altered patterns of information processing.

#### Strengths and Limitations of Resting-State MEG Studies in BD Population

Although of important value, the resting-state MEG study by Chen et al. (142) exploring long-range synchronization had a number of limitations. For instance, although significant information can be gathered by exploring euthymic patients (i.e., with no overt depressive or manic symptoms), the pool of subjects was relatively small (*n* = 10). Moreover, in addition to being conducted in sensor space, the authors focused on the frontal cortex activity, with oscillatory modulations of only 11 of their 306 MEG channels being reported. The investigation of the neural network connectivity pattern of BD using MEG is clearly still in its early days, and more studies are needed to elucidate the key connectivity patterns that define this illness.

### Summary of MEG Findings

**Figure 1:**
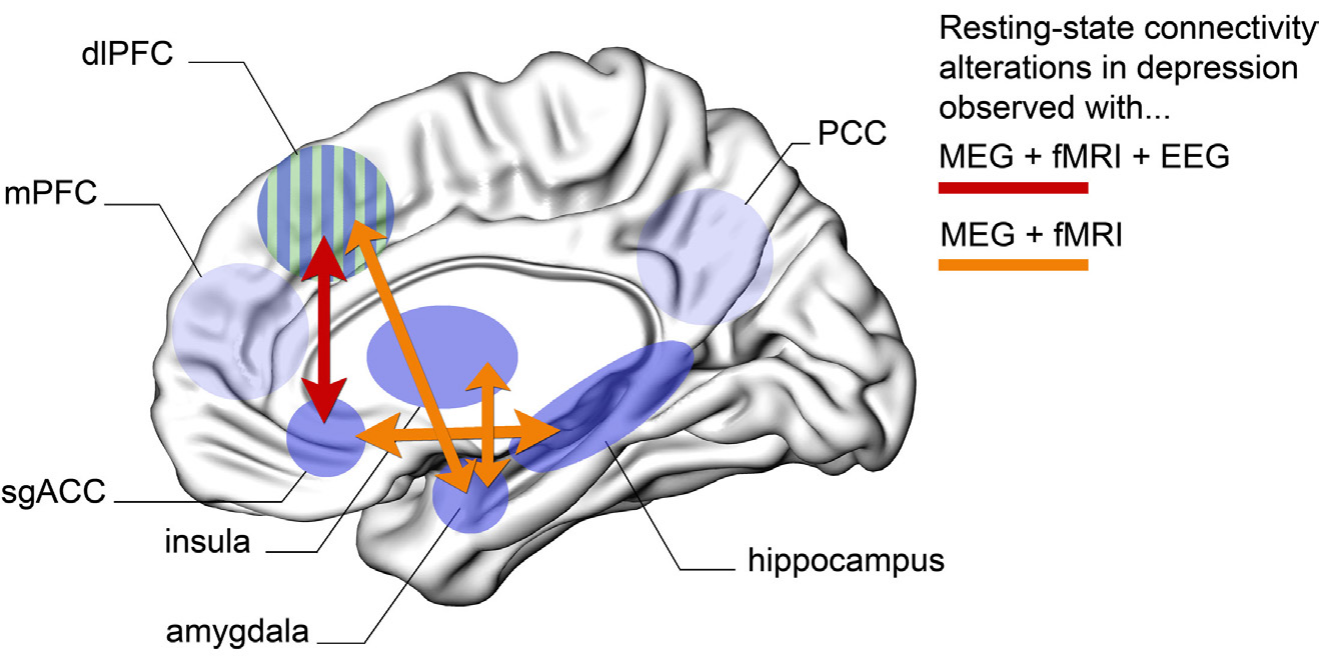
**Schematic overview of the key brain regions that show abnormal long-range connectivity patterns in subjects with MDD**. Here, we only show areas for which evidence has been confirmed across at least MEG and fMRI modalities. Orange arrows represent altered connection between two brain regions that has been confirmed using both MEG and fMRI resting-state paradigms. Red arrow represents altered resting-state connectivity between two regions that has been confirmed across MEG, EEG, and fMRI. Abbreviations: MEG, magnetoencephalography; EEG, electroencephalography; fMRI, functional magnetic resonance imaging; dlPFC, dorsolateral prefrontal cortex; mPFC, medial prefrontal cortex; sgACC, subgenual anterior cingulate cortex; PCC, posterior cingulate cortex; MDD, major depressive disorder. (Green–blue striped area represents dlPFC shown here from a medial view perspective for convenience.)

The present overview shows that MDD individuals have enhanced connectivity patterns within nodes of the DMN (as evidenced by resting-state fMRI and EEG studies), as well as altered connectivity between areas of the DMN and the limbic system (particularly between subgenual ACC and hippocampus, as evidenced by fMRI and MEG). Moreover, there is evidence for hypoactivity between regions of the CEN and the limbic system (particularly dorsolateral PFC and amygdala, as observed through fMRI and MEG), alterations between the nodes of the DMN and the CEN (particularly hyperconnectivity between the subgenual ACC and the dorsolateral PFC, as noted using fMRI, EEG, and MEG) and, finally, hyperconnectivity between the insula and the limbic system (amygdala, as noted by fMRI and MEG studies). Overall, projections from and to the subgenual ACC, as well as the dorsolateral PFC, appear to be critical in the treatment and expression of depressive symptoms. Figure 1 illustrates the key patterns of altered long-range connectivity in MDD patients, observed using both MEG and fMRI (and in some cases also with EEG).

Most of the work in BD arises from resting-state fMRI research, which has observed altered connectivity between areas of the DMN and the limbic system (notably between the mPFC and the amygdala), hyperconnectivity between nodes of the DMN and CEN (particularly between the medial PFC and dorsal/ventrolateral PFC), and hyperconnectivity between the ventrolateral PFC and the amygdala. Across all three neuroimaging modalities of fMRI, EEG, and MEG, altered (mainly decreased) connectivity in the PFC has been noted. This hypoactivity in the frontal lobe has been thought to be due to the presence of psychotic symptoms in a proportion of BD patients (120). Hence, overall, the amygdala appears to be a key region in BD. Given the scarcity of resting-state connectivity studies in the BD population, we could not illustrate by a figure the intrinsic patterns affected in this pathology. This exemplifies the important need of conducting more resting-state MEG studies to support (and further characterize) the functional findings that are observed through an MRI in BD. Such electrophysiological-based results, especially if conducted in source space, could immensely improve our understanding of the fundamental network disorganization of this pathology.

## Challenges, Pitfalls, and Methodological Recommendations for Future Studies

The assessment of resting-state connectivity patterns in psychiatric populations with MEG is a fairly new field still faced with substantial technical challenges. This section addresses the most important methodological issues that need to be understood and taken into account. Most importantly, in addition to delineating current limitations, we provide suggestions and methodological recommendations to help the field move forward.

### Choosing an Appropriate Connectivity Metric: Lack of a Gold Standard

In contrast to functional connectivity estimations in fMRI, where the primary measure is straightforward correlation between the BOLD time series in various voxels or ROIs, MEG connectivity estimation is a more complex endeavor (146). This complexity has two distinct causes: first, many common connectivity metrics come with important methodological pitfalls, and second, the richness and multifaceted nature of neuromagnetic signals can allow the exploration of a wide variety of interactions (e.g., phase-amplitude coupling, phase-phase coupling). A question that is thus reoccurring is the following: Which coupling method should one use? Choosing the right connectivity metric to assess long-range MEG coupling is a critical decision that can easily bias the results of the study.

Generally speaking, most of the commonly used interaction measures (e.g., coherence or phase-locking value) face limitations caused by linear mixing. This is a problem often referred to as field spread (MEG) or volume conduction (EEG) when dealing with sensor level data, or signal leakage when exploring source-level data (48). Although several coupling measures have been proposed [e.g., Ref. (47, 51, 147–149)], there is no consensus as to which one provides the best estimate of true cortical interaction. Ideally a reliable and robust measure would fulfill two criteria: it would be (a) minimally sensitive to linear mixing and (b) maximally sensitive to the specific physiological mechanisms that underlie the neural interaction. Indeed, there is not much use for a technique that is entirely immune to field spread effects if the quantity that it estimates does not capture the true physiological functional interaction. Without a clear hypothesis about the precise long-range physiological coupling mechanism, one compromise that may well be worth considering is to examine connectivity through a combination of complementary metrics. This could be achieved, for instance, through joint exploration of phase-based and amplitude-based measures. In this context, we encourage the assessment of phase-lag index (52, 66) in parallel to band-limited envelope correlations (149–151).

### Sensor- vs Source-Level Analyses

Although important information can be gained from combining sensor-level MEG data with advanced connectivity metrics, source-space network assessments are key to simultaneously identify the neuroanatomical substrates and functional role of the involved networks. Moreover, source-level estimation is critical to help bridge the gap between MEG and fMRI findings in the field of psychiatry. Although most of the studies reviewed here use source-space connectivity measurements (16, 132–134, 152, 153), most electrophysiological studies still conduct their analyses in sensor space. A question one might ask is which source estimation technique would be considered most efficient for the specific aim of measuring resting-state MEG source-level connectivity patterns. Most of the available techniques differ in their underlying assumptions about the properties of the sources (18, 154). Attempts to infer the most appropriate source reconstruction method based on the real data are hard to evaluate given that the ground truth is unknown. Using simulations can help us appreciate the strengths and limitations of a coupling method, but the extent to which it is useful for its application to real data is difficult to assess. The lack of a reliable gold standard is a concern for MEG analyses in general and for MEG resting-state network assessments in particular. One could argue that the discussion on identifying the best inverse method might be considered an ill-posed question in itself with no unique solution. Nevertheless, we expect most families of source estimation methods (e.g., minimum-norm or spatial filters) to provide similar results when applied properly. Above all, what is most important is to understand the pitfalls and limitations of a chosen method and their impact on source-space connectivity estimations (155).

### Stability of MEG-Based Resting-State Networks Estimations

A challenge that is not yet entirely resolved is that of the robustness and consistency of MEG-based resting-state estimation over time and across participants. Recent research has addressed the reliability of MEG resting-state connectivity metrics (156) and its test-retest reliability (157). Both intersubject and intrasubject consistency of MEG resting-state network estimations have been investigated, and it has been found that, while variability exists, seed-based and appropriate averaging techniques allow to compare subjects between and within groups (158). Epoch length is a potential source of variability that also needs to be considered when measuring resting-state connectivity [for an EEG study, see Ref. (159)]. Such parameters need to be carefully chosen prior to designing the resting-state MEG acquisition protocol. In addition, when it comes to clinical patients, it is recommended to acquire longer resting-state data than for healthy subjects as there is a higher risk of artifacts [see Contrasting Controls and Patients: Differences in Artifacts and Signal-to-Noise Ratio (SNR)]. The psychiatry-focused studies that were reviewed here used recording lengths that varied between 2 and 6 min, although most of them used 3–4 min. A gold standard for data length in MEG resting-state protocols is lacking. Three minutes seems to be an acceptable lower limit and 4–5 min can be considered a reasonable recommendation and likely necessary in the case of patient populations (where subsequent data loss is expected because of more artifacts). Similarly, there is currently no consensus on whether resting-state protocols should be performed with eyes open or closed. About half of the MEG resting-state studies reviewed here were carried out with eyes open and the other half with eyes closed. Because of the relatively low number of resting-state studies in MEG and because of different methodological constraints in MEG and fMRI, it seems too early to make a final decision. Given this, we would recommend acquiring both eyes open and eyes closed if possible. If this is not feasible, we suggest using eyes open with a fixation cross to minimize eye movements. Eyes closed resting state is associated with strong alpha power increases (which might in theory interfere with subsequent network analyses), and participants are at a higher risk of getting drowsy and potentially falling asleep during the recording.

### Contrasting Controls and Patients: Differences in Artifacts and Signal-to-Noise Ratio (SNR)

Comparisons between MEG resting-state connectivity patterns obtained in controls and patients are faced with additional difficulties caused by the pathological conditions. Increased head and body movement artifacts, eye blinks, and saccades in patient populations are not uncommon, and they all lead to poorer data quality compared with data acquired in healthy subjects. For equal MEG scanning durations, artifact rejection techniques will ultimately lead to less data being preserved for the patients, which may in turn yield lower SNR in patient data compared with controls. These differences in SNR must be avoided, or at least controlled for, since they will lead to differences in functional interaction patterns that may have nothing, or little, to do with the pathology at hand and rather reflecting differences in data quality. Minimizing data rejection through the use of artifact correction techniques such as ICAs could be of interest, although the differential application of ICA to the two groups (i.e., more extensive in the case of patient data) could also lead to differences that may bias connectivity findings and data interpretation. To address artifact-related SNR discrepancies between patients and controls, we recommend planning to acquire more data in patients from the start of the project or alternatively to use a subsample of data from the controls to achieve comparable SNR across the two groups.

A second, often overlooked, issue is that pathological changes in local signal amplitude can affect the estimation of long-range connectivity in patients and thereby lead to group differences that are in fact a reflection of inadequate or unreliable coupling estimation. This can occur because lower signal amplitudes (that equate noise levels) will *de facto* lead to lower SNR. A lower SNR within a given frequency band can affect, for instance, the estimation of phase. In such a case, the reduction or vanishing of a measure of interareal phase coupling, compared to controls, cannot be taken as an indication of connectivity break down, rather it is the result of poor phase estimation in patients due to lower local SNR. This phenomenon will also affect interareal cross-frequency phase-amplitude coupling. Overcoming such limitations is not trivial. A good rule of conduct is not to focus on interareal interaction measures alone, but to systematically calculate spectral power in the frequencies and nodes of interest. If the powers show statistically significant differences across the groups, one could attempt to randomly use subsamples of data to control for the effect of amplitude across the two groups (bootstrapping and stratification techniques could be useful here).

### Effect of Age and Medication on Connectivity Patterns in Psychiatry

In both healthy and pathological populations, age has been shown to be an important variable that can affect brain structure, cognitive functions, and connectivity patterns (160–162). At the anatomical level, volumes of cortical gray and white matter change with age. On the one hand, among neurotypically developed individuals, gray matter density of frontal and parietal lobes displays an inverted U pattern, with volume increasing until adolescence, then declining. However, this may not be the case of other brain regions (162, 163). On the other hand, white matter volume appears to steadily increase until around 30 years of age (162, 163). At the functional level, task-based studies in fMRI have observed focal increases in activity with age, for instance, in the dorsolateral PFC, ventrolateral PFC, and premotor cortex (164). Changes in connectivity between certain brain regions also seem to take place with age. Of note, long-range synchronizations that underline the processes of cognitive functioning (e.g., attention, working-memory, inhibition) appear to grow in strength until the third decade of life (161, 165). Compared to these findings in healthy cohorts, deficits observed in illnesses, such as schizophrenia, are found to be similar, albeit with steeper decline in some function, such as abstract thought [e.g., Ref. (160)].

Age of illness onset is also an important factor to take into consideration as early/preadolescence onset of psychopathologies typically correlate with worse prognosis and more severe clinical symptoms (166, 167). Moreover, in BD, early onset is seen to be linked to more comorbid disorders (e.g., anxiety, substance abuse), shorter euthymic periods, and more attempts of suicide (168, 169). Taken together, age is a critical factor when conducting connectivity analyses or correlations between symptoms and connectivity patterns, particularly in psychiatric population, to ensure that statements made about group differences are in fact due to true discrepancies between the evaluated cohorts and not due to an age effect (170).

Medication is also a variable for that has substantial effects on the neural network of psychiatric patients, with different types of pharmacotherapies impacting connectivity in distinct ways (e.g., selective seratonergic vs noradregenic reuptake inhibitor) (171). A review of longitudinal MRI-based studies noted that part of the gray matter volume decreases and ventricle enlargement in schizophrenia patients could be explained by cumulative exposure to antipsychotic treatment (172). In MDD, antidepressant treatment seems to modify the connectivity between the nodes of the DMN, as well as corticolimbic connectivity, at both rest and during affective tasks [e.g., Ref. (173, 174)]. However, other studies find the effect of psychotropics on functional connectivity to be inconclusive (175). Part of the difficulty in untangling the influence of treatment lies in the complexity of conducting longitudinal studies, which ideally include drug naïve patients who are either individuals at risk of developing a psychiatry illness or first-episode psychosis or mania patients, as well as chronic patients to compare with. A number of studies that have investigated birth cohorts [e.g., Ref. (176)] have enlightened the field the most as they take into account maximal information regarding context, neurodevelopmental factors, environmental influences, longitudinal notes on symptoms, and treatments effects.

Finally, it is important to note that non-medication drugs, such as nicotine and caffeine, also appear to alter resting-state networks in healthy and clinical populations. Evidence of this effect has been reported using fMRI (177–182) and MEG (183). Future connectivity studies should incorporate these variables in their analyses.

## Conclusion and Future Directions

This review is the first of its kind to examine the literature’s findings on resting neural network connectivity patterns of BD and major depression disorder, based on MEG studies. A global analysis of current scientific papers demonstrate that the two illnesses display functional abnormalities that affect the way information is integrated, locally, and transferred from one brain region to another through long-range connections. Moreover, this review illustrated that resting-state neuroimaging paradigms are a useful way to access the disorganized brains of individuals with psychopathologies. Finally, although still in its early days, MEG carries the potential to significantly advance our understanding of large-scale network alterations associated with psychiatric disorders (184).

Overall, the PFC, in particular the mPFC which is at the core of the DMN and of social cognition, is affected across both psychopathologies. Given that this brain region is one of the last to develop during neurodevelopment (185, 186), it is not surprising that most mental health issues arise during adolescence and that any early brain damage, detrimental environmental factor, or oxidative stress can affect a person’s personality, theory of mind, emotional maturity, empathy, and healthy resting neural wiring (187–189). Of note, among depressed individuals, patterns of dysfunctional connectivity are repeatedly observed across the three major neuroimaging modalities reviewed (fMRI, EEG, and MEG), particularly altered long-range connectivity between the DMN and the limbic system, as well as between the DMN and the CEN. In MDD population, the recurrent dysfunctional connectivity patterns involved the subgenual ACC. As for the BD literature, the most consistent findings stemmed from resting fMRI studies, where functional connectivity was altered between regions of the DMN and the amygdala in BD.

An explanation for the imbalance in the amount of scientific papers published in these two mood disorders could be that depression is the mental illness affecting the largest percentage of individual worldwide in its various forms (e.g., MDD, postpartum depression, seasonal onset depression), while BD is symptomatically more complex and heterogeneous. Thus, when interpreting neuroimaging results, researchers should consider the effect of additional psychological factors, such as manic/cyclic mood and history of psychosis (120), as well as medication when attempting to untangle the connectivity pattern affiliated with BD.

Resting-state MEG is expected to continue gaining momentum in psychiatry. One promising application is its ability to enhance the understanding of how neuromodulation (e.g., rTMS) can change the neural circuitry of mood disorder patients. Indeed, there is cumulating evidence that rTMS might reduce symptoms in MDD patients (190–193). Hence, exploring the different ways this tool changes resting-state networks after stimulation could help further elucidate connectivity patterns in these patients, and possibly lead to new neuromodulation targets for the treatment of MDD.

Our recommendations for future studies are to further explore the potential of examining functional and effective neural network connectivity in psychiatric disorders using a combination of tools, multimodal imaging techniques, yet employ common terminology (194). As far as MEG is concerned, performing the connectivity analysis in source space is highly recommended to improve the interpretability of the findings. In addition, the informed choice of the connectivity framework and network metrics is critical to avoid misinterpretations. The use of advanced methods such as graph metrics or machine learning as a data mining tool in this field is also a promising venue for future research. By doing so, a more complete picture of how mental illness affects information propagation can be acquired, thus allowing for the development of more efficient treatment for patients.

## Author Contributions

GA and KJ designed and wrote the review; GA, A-SH, EC, VM, DA, TT, and KJ conducted the literature research, the critical assessment thereof, and comprehensive proofreading of the manuscript.

## Conflict of Interest Statement

The authors declare that the research was conducted in the absence of any commercial or financial relationships that could be construed as a potential conflict of interest.

## Abbreviations

BD, bipolar disorder; EEG, electroencephalography; fMRI, functional magnetic resonance imaging; MDD, major depressive disorder; MEG, magnetoencephalography; PFC, prefrontal cortex; CEN, central executive network; ACC, anterior cingulate cortex; DBS, deep brain stimulation.
